# Increased Endogenous Nitric Oxide Release by Iron Chelation and Purinergic Activation in the Rat Carotid Body

**DOI:** 10.2174/1874091X00701010001

**Published:** 2007-06-15

**Authors:** Man-Lung Fung, Meifang Li, Sukhamay Lahiri

**Affiliations:** Department of Physiology, University of Hong Kong, Pokfulam, Hong Kong, China

**Keywords:** Carotid chemoreceptor, HIF, hypoxia, NO synthase, purinergic receptor

## Abstract

We examined the hypothesis that hypoxic chemotransduction with stabilization of HIF-1 and activation of purinoceptors stimulate the endogenous NO production in the rat carotid body. The effects of blockade of purinoceptors with suramin, or blockade of HIF-1α hydroxylation by suppressing prolyl hydroxylase (PAH) activity on the endogenous NO release measured electrochemically by microsensor inserted into the isolated carotid body superfused with bicarbonate-buffer were examined. Suramin did not change the resting NO level under normoxic conditions but it significantly decreased the hypoxia-induced NO elevation in a dose-dependent manner. Suramin (100μM) blocked the NO response to acute hypoxia by 53%. Intracellular iron chelator, ciclopirox olamine (CPX) significantly increased the resting NO release close to the hypoxic level, which was reversed by FeSO_4_ or blocked by L-NMMA. Also, PAH inhibition with dimethy-loxalylglycine (DMOG) moderately increased the resting NO release. In the presence of CPX and DMOG the resting NO release was increased to the hypoxic level. Collectively, results suggest that iron chelation and purinoceptor stimulation play a role in the hypoxic chemotransduction for an increase in the endogenous NO production in the rat carotid body.

## INTRODUCTION

Hypoxic chemotransduction of the carotid body is central to the regulation of cardiorespiratory performance by the carotid chemoreflex. Type-I glomus cells in the carotid body are believed to be the oxygen-sensitive sites for the chemotransduction [1]. In addition to the membrane-limited mechanism with modulation of potassium channel activities by hypoxia [2, 3], recent evidence support an involvement of stabilization of hypoxia-inducible factor (HIF)-1 in the chemotransduction [4]. HIF-1 is a heterodimeric complex consists of two subunits. The protein level of HIF-1α subunit is post-transcriptionally regulated by prolyl and asparaginyl hydroxylase (PAH) activity for the HIF-1α hydroxylation in the presence of oxygen for the proteasomal degradation of HIF-1α with ubiquitylation [5, 6]. The HIF-1β subunit is constitutively expressed independent of oxygen level. Depletion of cofactors required for the PAH activity, physiologically by hypoxia or pharmacologically by intracellular iron chelation, increases the HIF-1α protein level in the carotid body [7–9]. In addition, oxoglutarate analogue suppresses PAH activity and increases endogenous HIF-1α level [10]. Roy *et al*. [11] reported that intracellular iron chelator, ciclopirox olamine (CPX) inhibits potassium currents and increases cytosolic calcium level in rat glomus cells with increased carotid chemoreceptor activity.

In addition to catecholamines [1, 12], recent studies suggest ATP, which is co-released with acetylcholine, as a putative neurotransmitter involved in the chemotransduction [13, 14]. Purinergic transmission is indicative because of the expression of purinoceptor subunits P2X2 and P2X3 in the nerve fibers co-localized with glomus clusters containing tyrosine hydroxylase [15]. Importantly, ATP excited petrosal neurons *via* ionotropic P2X receptor [16]. Also, P2X purinoceptor suramin inhibited hypoxic transmission of the carotid body [16]. Thus activation of purinergic transmission plays a role in the chemotransduction.

The hypoxic response of the carotid body is modulated by nitric oxide (NO), presumably produced by NO synthase (NOS) located in nerve endings and vascular endothelium in the carotid body [17–22]. Hence, NOS inhibition enhances the hypoxic response of the carotid body despite minimal effects on the resting activity [23–25]. We have shown an increase in endogenous NO level in the rat carotid body in acute hypoxia, which is physiologically important to the inhibition of carotid chemoreceptor activity for a negative feedback control of the activity in hypoxia [26, 27]. At present, there is a paucity of information on the physiological events between the chemotranduction and the endogenous NO release in the carotid body. It was hypothesized that hypoxic chemotranduction with stabilization of HIF-1 and activation of P2X/P2Y purinoceptors stimulate the endogenous NO production in the rat carotid body. Hence, the aims of the present study were to determine the effects of blockade of purinoceptors with suramin, or suppression of PAH activity with iron chelator CPX, and with the oxoglutarate analogue dimethyloxalylglycine (DMOG), on the endogenous NO release produced in some intraglomic structure, probably chemoreceptor cells and efferent fibers.

## MATERIALS AND METHODOLOGY

### Isolation of the Carotid Body

The experimental protocol for this study was approved by the Committee on the Use of Live Animals in Teaching and Research of The University of Hong Kong. Following deep anesthesia with halothane, young adult Sprague-Dawley rat (*ca.* 150-200 g) was decapitated and the carotid bifurcation was excised rapidly. The carotid body was carefully dissected free from the bifurcation in chilled rat Ringer’s solution oxygenated with 95% O_2_ and 5% CO_2_. The carotid body was then incubated in a tissue bath with collagenase (0.06%) and protease (0.02%) in oxygenated Ringer’s for 30 min at 35±1 °C. The carotid body was then held in the recording chamber at 35±1 °C, and superfused with oxygenated Ringer’s solution in a flow rate of 2 ml/min.

### Nitric Oxide Electrode

The fabrication and calibration of the NO electrode has been described in previous reports [26–28]. Briefly, a laboratory-made Pt wire insulated in a poly-ethylene tube was dipped in Nafion. The Nafion-coated electrode was further modified with palladium and iridium oxide particles for improving the sensitivity of the NO electrode. Then, a thin film of poly-*o*-aminophenol (POAP) was deposited in the outer layer to ameliorate the selectivity of the NO electrode and to avoid fouling by proteins. NO standards were prepared by serial dilution of a saturated NO solution. The saturated NO solution was prepared by bubbling phosphate buffered saline (pH 7.0) with pure N_2_ for 30 min to remove O_2_, following by NO gas (Matheson) for 30 min. Standards were made fresh for each experiment and kept in a glass flask with a rubber septum. Electrochemical experiments were performed on a CHI 660A Electrochemical Analyzer (CH Instruments, USA) at room temperature in a three compartment cell with a Ag/AgCl reference electrode, a Pt wire auxiliary electrode and a chemically modified electrode as working electrode. The NO electrode was calibrated with successive injections of various concentrations of NO from 20-1000 nM to the Ringer’s solution in the recording chamber. The current was measured at a voltage of 0.85 V. The current response to various NO concentrations in nanomolar range was very close to linear with the coefficient of the linear equation (y = a + 	bx) not less than 0.95. The detection limit of our NO electrode was about 10 nM with signal to noise ratio of 3. The current responses to NO were comparable in Ringer’s solution gassed with 95% O_2_ / 5% CO_2_ or 95% N_2_ / 5% CO_2_ in the presence or absence of dopamine (30 nM). The NO electrode responded to dopamine in micromolar (1 μM) but not in nanomolar concentration ranges (30 nM) and the current response was more selective for NO than dopamine with a ratio of about 1:100. The current response of the NO electrode was examined before and after the experiment.

### Experimental Paradigm

The electrode and carotid body were equilibrated in the perfusate for 15-30 min. The tip of the NO electrode was gently inserted into the carotid body under visual guidance with a dissecting microscope. Following current recording in the resting condition for 10-15 min, acute hypoxia was induced for 5 min following by recovery for 20-30 min. Acute hypoxia was induced by switching the perfusate to Ringer’s solution gassed with 95% N_2_ and 5% CO_2_. To assess the involvement of HIF-1 and purinergic transmission, the effects of blockade of P2X/P2Y purinoceptors with suramin, or suppression of PAH activity with CPX, DMOG on the endogenous NO release in the superfused rat carotid body were examined. The effect of CPX can be reversed by FeSO_4_ [11] and the endogenous NO production was blocked by non-selective NO synthase inhibitor N^G^-Monomethyl-*L*-arginine acetate (L-NMMA). For the drug treatment, L-NMMA (100 μM) or suramin (10-100 μM) or CPX (5 μM) or DMOG (1 mM) or FeSO_4_ (200 μM) or catalase (1000 unit/ml) or superoxide dismutase (600 unit/ml) was added freshly to the Ringer’s solution for the perfusion.

### Materials and Pharmacological Agents

The rat Ringer’s solution contained (mM): NaC1 125, KC1 3.1, NaHCO_3_ 26, NaH_2_PO_4_ 1.25, MgSO_4_ 1.3, CaCl_2_ 2.4, D-Dextrose 10, pH at 7.35. K_2_IrCl_6_, K_2_PdCl_6_, o-Aminophenol and Nafion perfluorinated ion-exchange resin in alcohol were obtained from Aldrich. Chemicals were obtained from Sigma.

### Data Analysis

For the measure of NO release, the resting and peak values of the current were subtracted and calibrated in NO concentration (nanomoles) according to the current response curve of the NO electrode. Values were normalized as a percentage of control if necessary and presented as mean+/-SE. Means were compared with Student *t*-test or the non-parametric Wilcoxon signed-rank test. ANOVA with a post-hoc test (Dunnett’s *t*-test) was used for multiple comparisons of values in studies among groups. Differences were considered significant at *P*<0.05.

## RESULTS

**Fig. (1):**
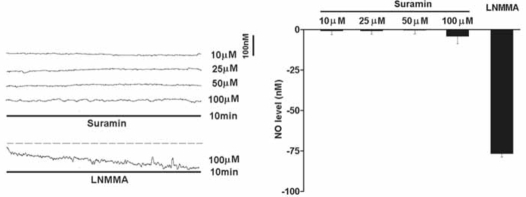
Under normoxic conditions, perfusion of suramin did not significantly reduce the endogenous NO release measured electrochemically by microsensor inserted into the rat carotid body superfused with Ringer’s solution. Left panel shows the current tracings with suramin (10, 25, 50 and 100μM) or L-NMMA for 10 min. Vertical bar is the calibrated NO concentration. Right panel shows the summary of the data. The resting NO level was significantly reduced by L-NMMA but not by suramin (n=6-10 carotid bodies in each group). The dashed line depicts the virtual baseline for the measurement in a tracing.

The resting NO level decreased gradually with the perfusion of a non-selective NOS inhibitor L-NMMA (100μM) for 10 min under normoxic conditions (Fig. 1). On average, the endogenous NO release was reduced by 76.7+/-2.1nM (*n*=6) in the carotid body. However, perfusion of suramin (10-100μM) did not significantly reduce the resting NO release under normoxic conditions (Fig. 1).

**Fig. (2):**
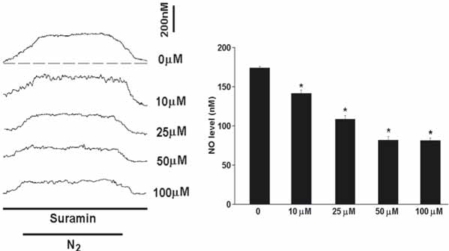
Perfusion of suramin significantly decreased the hypoxia-induced NO elevation. Left panel shows the current tracings in acute hypoxia (N_2_, bar 5 min) with or without the presence of suramin. Vertical bar is the calibrated NO concentration. Right panel shows the summary of the data. The NO level was significantly reduced by suramin in a dose-dependent manner (n=6-10 carotid bodies in each group). The dashed line depicts the virtual baseline for the measurement in a tracing.

The NO level rapidly elevated to a plateau level within minutes in acute hypoxia (Fig. 2). On average, the NO release was elevated by 174+/-2.3nM in the carotid body in hypoxia (*n*=10). Suramin (10-100μM) significantly decreased the hypoxia-induced NO elevation in a dose-dependent manner (Fig. 2). On average, suramin at 100μM reduced the NO release to 82+/-3.4nM (*n*=6) and blocked the hypoxic response by about 53% (*n*=6).

**Fig. (3):**
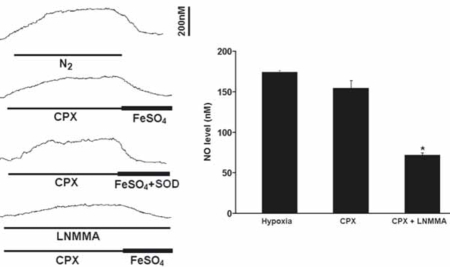
Perfusion of CPX significantly increased the resting NO release under normoxic conditions. Left panel shows the current tracings from top to bottom in acute hypoxia (N_2_, *bar* 5 min), in the presence of CPX followed by FeSO_4_ or by FeSO_4_ with superoxide dismutase (SOD) during resting or in the presence of L-NMMA. Vertical bar is the calibrated NO concentration. Right panel shows the summary of the data. The resting NO level was significantly increased by CPX to a level close to the level induced by hypoxia and the NO level was significantly blocked by L-NMMA (n=6-10 carotid bodies in each group).

The perfusion of CPX (5μM) alone at resting significantly increased the NO level by 154.3+/-9.8nM (*n*=6), which was slightly lower than the level induced by hypoxia (Fig. 3). In addition, the CPX-induced NO elevation was significantly reversed by FeSO_4_ and was attenuated by L-NMMA (Fig. 3). The effect of FeSO_4_ on the NO elevation was not altered by concomitant application of superoxide dismutase (*n*=5) or catalase (*n*=3) and the NO levels were fully recovered to the resting levels (Fig. 3).

**Fig. (4):**
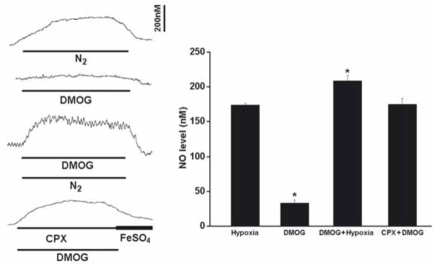
Perfusion of DMOG increased the resting NO release and the hypoxia-induced NO elevation. Left panel shows the current tracings from top to bottom in acute hypoxia (N_2_, *bar* 5 min), in the presence of DMOG during normoxia or hypoxia, in the presence of DMOG and CPX in normoxia. Vertical bar is the calibrated NO concentration. Right panel shows the summary of the data. The resting NO level was increased by DMOG but the level was significantly less than that induced by hypoxia. DMOG also increased the NO release during hypoxia. In the presence of CPX and DMOG the resting NO release was increased to the hypoxic level (n=6-10 carotid bodies in each group).

During the perfusion of DMOG (1mM), the resting NO release was increased by 33.7+/-4.7nM (*n*=6), although the elevated NO level was significantly less than that induced by hypoxia (Fig. 4). DMOG increased the hypoxia-induced NO elevation and CPX significantly increased the NO release induced by DMOG (Fig. 4).

## DISCUSSION

This is the first study reporting an increase in endogenous NO production mediated by iron chelation and purinergic transmission in the carotid body. The major observations of this study are that: (1) iron chelation, which could block HIF-1α hydroxylation by suppressing PAH activity under normoxic conditions, closely mimic the effect of acute hypoxia on the NO production in the carotid body; (2) blockade of P2X and/or P2Y purinoceptors attenuated the effect of acute hypoxia on the NO production in the carotid body. Hence, CPX and DMOG rapidly elevated resting NO release in normoxia to levels comparable to the hypoxic level in the carotid body. In addition, suramin did not change the resting NO release under normoxic conditions but dose-dependently decreased the hypoxia-induced NO elevation in the carotid body. Taken together, these results suggest that iron chelation and purinoceptor activation play a role in the hypoxic chemotransduction for an increase in the endogenous NO production in the rat carotid body.

In consistent with our previous reports [26, 27], the NO level elevated rapidly within a minute of hypoxia and it reached its peak level in 4-5 min of hypoxia when O_2_ concentration in the bath was reduced to its nadir (about 1 mmHg). Then, the NO concentration decreased gradually back to the resting level during recovery to normoxia from hypoxia (Fig. 2). The fact that blockade of NOS activity decreased the basal level of NO and largely attenuated the release of NO in hypoxia, suggests that the endogenous NO production is specific to the NOS in the carotid body.

The observations that under normoxic conditions CPX and DMOG increased the endogenous NO level close to the level induced by acute hypoxia, indicate that HIF-1 activation may be involved in the endogenous NO release during acute hypoxia. Results lend support to the idea that HIF-1 stabilization could play a role in the chemotransduction during acute hypoxia. Neither the glomic structure nor the mechanisms underlying are known, but there are data suggesting an activation of chemotransduction by HIF-1 in the chemosensitive cells. Hence, CPX increases the HIF-1α level and mimics the hypoxic effects on the potassium current, intracellular calcium level in the glomus cell and carotid chemoreceptor activity [7, 8, 11] as well as the NO release shown in this study. In addition, the CPX-induced NO elevation was normalized by iron supplement with FeSO_4_, which cannot be attributed to the generation of superoxide anion because superoxide dismutase or catalase did not altered the FeSO_4_ action. Moreover, suppression of PAH activity with DMOG also increased the resting NO release, despite a weak effect comparing with that of the CPX. In this context, it has been reported that DMOG alone had minor effect on HIF-1α level under normoxic conditions but the effect was prominent in ischemic conditions in the mouse skeletal muscle [10], which is consistent with the findings in this study. Yet, DMOG is a non-specific 2-oxoglutarate-dependent dioxygenase inhibitor that may have non-HIF targets [29]. Also, it has also been shown that inhibition of HIF hydroxylation in carotid body slices with 1 mM DMOG had no effect on the exocytotic response of the cell to hypoxia [30]. Thus, it is plausible that a separate mechanism may be mediated by iron chelation. Indeed, it has been proposed that Fenton reaction and free radicals may be involved in the hypoxic chemotransduction, which may be interfered by iron chelation [11, 30].

The observations that suramin did not change the resting NO level but dose-dependently attenuated the hypoxia-induced NO elevation, suggest that activation of purinergic receptor is involved in the endogenous NO release during acute hypoxia. It is plausible that the NO production is increased by activation of ionotropic purinoceptors localized at the nerve endings apposed to the glomus cells in the carotid body. Studies have shown the expression of purinoceptor subunits P2X2 and P2X3 in the nerve fibers co-localized with the glomus clusters [15, 31]. In addition, NOS is localized in the nerve endings and plexus of nerve fibers in lobules of parenchyma cells and near small blood vessels of the carotid body, which plays physiological role in the NO inhibition of carotid chemoreceptor activity [25, 26, 32, 33].

For the NO production by NOS, L-arginine, NADPH and O_2_ are co-substrates. It is generally accepted that concentrations of L-arginine and NADPH are high in the tissues and are unlikely to be the limiting factor. Although the low level of O_2_ during hypoxia may reduce the NO synthesis in the catalytic reactions [34], it may not be the dominant factor *in vivo* because the residue amount of O_2_ in tissue during hypoxia may minimize the reduction in NO synthesis. In fact, it has been shown that NO concentration in brain tissue increases during cerebral ischemia [35], suggesting the O_2_ is taken from the tissue for the conversion of L-arginine to N^ω^-hydroxyarginine. Thus, O_2_ concentration may not be the only factor that determines the amount of NO production in the carotid body during hypoxia. In addition, activation of NOS is driven by calcium-dependent mechanisms. Elevation of cytosolic calcium concentration to about 400 nM is required for the binding between calmodulin and the NOS, which is necessary for the enzyme to become fully active [36, 37]. During hypoxia, elevation of intracellular calcium in the chemosensitive glomus cell is essential for the chemotrans-duction. In fact, a recent study has demonstrated morphological evidence supporting an increase in NO production in the glomus cell during acute hypoxia [38]. Thus, the NO released by the glomus cell could contribute to the hypoxia-induced NO elevation in the carotid body. In fact, increased HIF-1α level by iron chelators or PAH suppression has been shown to mimic the hypoxic effects on the potassium current, intracellular calcium level in the glomus cell and carotid chemoreceptor activity [7, 8, 11].

Moreover, increased autonomic efferent activity may be associated with an increase of cytosolic calcium concentration in the nNOS-containing efferent nerve fibers for the NO release in the carotid body. In this context, Campanucci *et al*. [39] recently reported that P2X receptors are localized to the efferent nerve endings and activation of these receptors by ATP could lead to an inhibition of chemoreceptor activities *via* an NO mechanism. Furthermore, P2X receptors are also localized to the afferent nerve endings apposed to the glomus cluster [31]. Thus, an increase in afferent activity during hypoxia could cause the calcium influx stimulating the NO production. We cannot rule out the NO released from the blood vessels given the fact that P2X receptors is expressed in endothelial cells [40, 41], although it is not clear the ex-tracellular ATP levels and the contribution of the endothelial P2X receptors to the NO elevation under hypoxic conditions. Thus, it is likely that there are multiple sources of NO, which could contribute to the control of the chemoreceptor activity.

It is well known that NO inhibits carotid chemoreceptor activity during hypoxia [23–25]. The increase in endogenous NO level in the rat carotid body in acute hypoxia is physiologically important to the negative feedback control of the carotid chemoreceptor activity in hypoxia [26, 27]. In fact, NO inhibits L-type voltage-gated calcium channels in glomus cells [42], and this could reduce the calcium entry, excitability and neurotransmitter release of the glomus cells during hypoxia. It is plausible that the elevated NO level could serve as a negative modulator to regulate the ATP release during acute hypoxia. In addition, it has been reported that NO can modulate the level of HIF-1α [43] *via* the phosphatidylinositol 3-kinase pathway [44] or by inhibiting the activity of prolyl hydroxylases under normoxic or hypoxic conditions [45]. Thus, NO could also be an important modulator regulating the HIF-1α level and HIF-1 activation in the carotid body.

In conclusion, iron chelation, which could suppress the PAH activity for HIF-1α hydroxylation under normoxic conditions, closely mimic the endogenous NO production in the carotid body during acute hypoxia. In addition, the hypoxia-induced NO production was reduced by blockade of P2X/P2Y purinoceptors in the carotid body. Thus, these results support that iron chelation and activation of purinoceptors play a role in the hypoxic chemotransduction for an increase in the endogenous NO production in the rat carotid body.
